# Research Progress on Two-Dimensional Layered MXene/Elastomer Nanocomposites

**DOI:** 10.3390/polym14194094

**Published:** 2022-09-29

**Authors:** Hailan Kang, Lishuo Han, Shule Chen, Shuao Xie, Mengjiang Li, Qinghong Fang, Shaojian He

**Affiliations:** 1 College of Materials Science and Engineering, Shenyang University of Chemical Technology, Shenyang 110142, China; 2 Key Laboratory for Rubber Elastomer of Liaoning Province, Shenyang University of Chemical Technology, Shenyang 110142, China; 3 Beijing Key Laboratory of Energy Safety and Clean Utilization, North China Electric Power University, Beijing 102206, China

**Keywords:** MXene, rubber, thermoplastic elastomer, nanocomposite

## Abstract

Two-dimensional (2D) transition-metal carbon/nitrogen/carbon nitride (MXene) has extremely high conductivity and easily modifiable surface functional groups. Compared with graphene, another 2D layered material, MXene is easily dispersed in water owing to its hydrophilic groups. Its unique characteristics make MXene a valuable material. Nanocomposites can be endowed with functionality when MXene is compounded with an elastomer. Particularly in electromagnetic interference shielding and sensing, MXene exhibits extraordinary properties. We review various preparation methods, properties, and applications of MXene and MXene/elastomer nanocomposites and present a summary of the prospects for MXene/elastomer nanocomposites, which are in their initial stage of development and providing promising results.

## 1. Introduction

Elastomer is a type of polymer that can approximately recover its original state immediately after being deformed. It is extensively employed in fields such as general production, advanced technologies, and defense. Elastomers, especially rubber, have low strengths and moduli, and nanofillers can be added to increase their strengths [1]. Nanofillers are compact but can have large surface areas. In addition, they are light and can achieve favorable mechanical properties, thermal stability, and dimensional stability with low filler content compared with microfillers [2]. With the rapid development of science and technology, functional elastomers are being increasingly employed in various fields, and nanofillers can endow elastomers with functionality, improve the transmission of electrons and phonons, and restrict quality transfer [3]. Various nanofillers are being applied to elastomers, such as carbon materials, including conductive carbon black, carbon nanotubes (CNTs), graphene, nanosilica, metal oxides, hexagonal boron nitride, layered double hydroxide, transition metal dichalcogenide, and 2D transition metal carbon/nitrogen/carbon nitride (MXene) [2,3,4,5]. Elastomers have shown diversity after composition with one or more nanomaterials [6,7,8].

**Figure 1:**
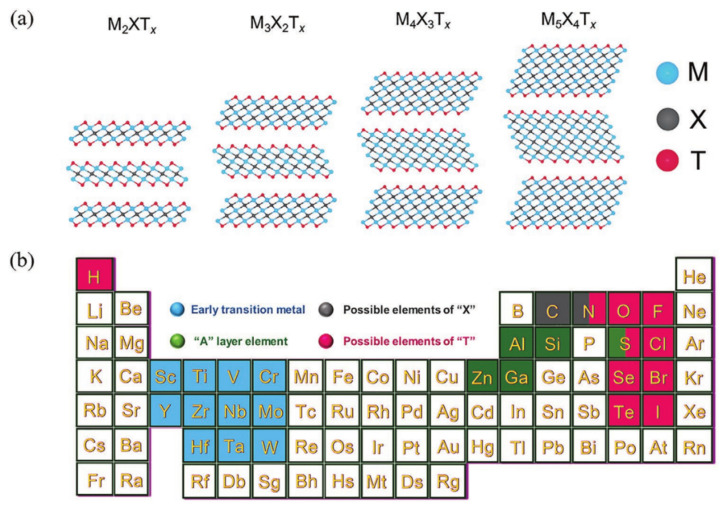
(**a**) Schematic structure of MXenes. (**b**) M, A, X, and T elements for MAX and MXene (Reprinted with permission from Ref. [28]. Copyright 2021, copyright Wiley-VCH).

MXene has attracted research interests in various fields since its introduction in 2011 [9]. The chemical formula of its family of 2D layered materials is M*_n_*_+1_X*_n_*, where M represents early transition metals, X can be C and/or N, and *n* = 1–4 (Figure 1a,b) [10]. MXene is obtained by removing layers A from three-dimensional (3D) layered ceramic materials of MAX phase by chemical etching. The corresponding chemical formula is M*_n_*_+1_AX*_n_*, where A is a main group element, such as Al and Si [11,12,13,14]. Common MXenes include Ti_3_C_2_T_x_ and Ti_2_CT_x_, where T_x_ denotes surface functional groups, such as -OH, -F, and =O. Given the electronegative layered structure and surface functional group -OH, it can easily be dispersed in water and is more convenient for surface modification and assembly [15,16,17] compared with graphene-based 2D nanomaterials. The conductivity of MXene is comparable to that of graphene. When the MXene surface contains more groups, it can exhibit semiconductor properties, and when fewer surface functional groups are available, MXene is relatively pure, exhibiting metal conductivity [18]. For example, the conductivity of MXene can reach up to 20,000 S·cm^−1^ after obtaining a thin film using the common Ti_3_C_2_T_x_ MXene aqueous dispersion. The maximum starting temperature of oxidation for Ti_3_C_2_T_x_ monolayer nanosheets is approximately 450 °C in air, with complete oxidation at 900 °C [19]. In addition, MXene has a high specific surface area and mechanical flexibility. Owing to its diverse characteristics, MXene can be suitably applied in electromagnetic interference (EMI) shielding and microwave absorption [20,21], sensors [22], energy storage [23], flame retardants [24], catalysis [25], adsorption [26], biomedicine [27], and other areas.

**Figure 2:**
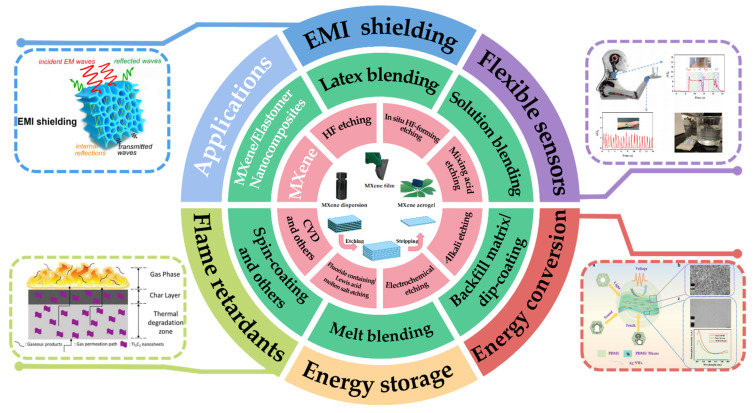
Available preparation methods for MXenes and preparation methods for and applications of MXene/elastomer nanocomposites. (The image of EMI shielding: Reprinted with permission from Ref. [16]. Copyright 2019, copyright Elsevier. The image of flexible sensors: Reprinted with permission from Ref. [29]. Copyright 2021, copyright Wiley-VCH. The image of energy storage and conversion: Reprinted with permission from Ref. [30]. Copyright 2021, copyright Elsevier. The image of flame retardants: Reprinted with permission from Ref. [31]. Copyright 2019, copyright Elsevier).

Owing to its excellent electrical conductivity, hydrophilicity, and 2D layered structure, MXene can be compounded with elastomers, such as natural rubber (NR), polydimethylsiloxane (PDMS), and thermoplastic polyurethane (TPU), to produce functional nanocomposites. In this paper, the preparation methods of MXene and MXene/elastomer nanocomposites are described, and research progress on the properties and applications of these nanocomposites is reviewed (Figure 2).

## 2. Preparation of MXenes

### 2.1. Hydrofluoric Acid Etching

**Figure 3:**
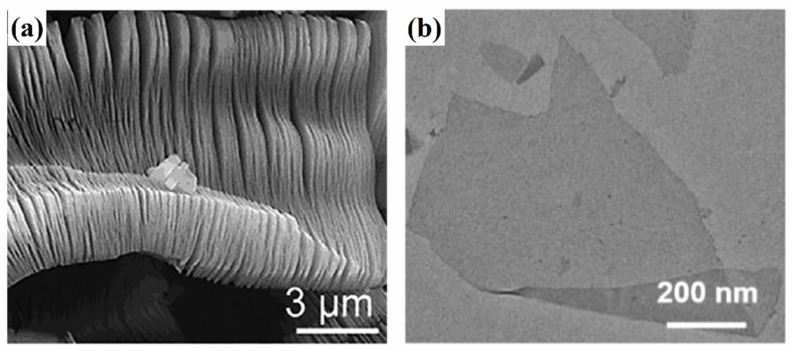
(**a**) Scanning electron microscopy image of the accordion-like multilayer MXene structure (Reprinted with permission from Ref. [34]. Copyright 2012, copyright ACS). (**b**) Transmission electron microscopy image of single-layer MXene nanosheets (Reprinted with permission from Ref. [35]. Copyright 2020, copyright Elsevier).

In 2011, Nguib et al. [9] found that aluminum in Ti_3_AlC_2_ can be dissolved completely when Ti_3_AlC_2_ of a MAX phase reacts with hydrofluoric acid (HF), occasionally obtaining Ti_3_C_2_T_x_, which was the first 2D MXene to be synthesized while searching for substances resistant to HF corrosion. As a common method, HF etching mixes MAX phase with HF, obtaining MXene by etching elements A. A higher HF concentration results in a shorter reaction time but higher toxicity and stronger corrosion, which is inconvenient for production and results in numerous structural defects in the materials. In addition, the interface state and hydrophilicity of materials can be altered by excessive -F. After etching, the excess acid and by-products should be cleaned and the pH should be regulated to approximately 6 by separating the multilayer MXene from the acid solution by repeated centrifugal washing. MXene exhibits an accordion-like multilayer structure (Figure 3a), and single-layer MXene (Figure 3b) is generally used for experimental materials. However, it is difficult to strip monolithic MXene mechanically, it being necessary to insert an intercalation agent between layers for ultrasonic stripping and delamination. Organic solvents are often used as intercalators, such as dimethyl sulfoxide [32] and tetramethylammonium hydroxide [33], resulting in slices with approximate thicknesses ranging from hundreds of nanometers to several micrometers.

### 2.2. In Situ HF-Forming Etching with HCl/LiF

For in situ HF-forming etching [36], hydrochloric acid (HCl) and lithium fluoride (LiF) are mixed and stirred for reaction with a MAX phase, and HF is generated in situ on the surface of the MAX phase to prepare a low-concentration etchant of the A phase. In situ HF-forming etching allows delamination peeling without the addition of an intercalator, as Li^+^ serves as an intercalator [37]. HF-forming etching can be subdivided into the clay method and the minimally intensive layer delamination (MILD) method, according to fluoride and hydrochloric acid concentrations. In early in situ HF-forming etching, HCl and LiF are mixed to etch Ti_3_AlC_2_, obtaining clay-like Ti_3_C_2_T_x_ in the so-called clay method. This method only needs an ultrasonic treatment of MXene for stratification, resulting in a small monolithic MXene but with several defects and oxidation. Furthermore, MILD [37,38] is an optimized method that increases the ratio of LiF to MAX to more than 7.5 and the HCl concentration from 6 to more than 9 mol·L^−1^. In addition, it does not need ultrasonication during delamination and provides a large monolithic MXene by shaking hands while avoiding oxidation and exhibiting fewer defects and higher quality. Li^+^ and H^+^ are important for MILD. Li^+^ is a restricting reagent, and excess HCl exchanges H^+^ with Li^+^ in the lamellar structure, leading to expansion and enabling stratification by shaking hands. Except for the commonly used LiF, other fluorides are used for in situ HF-forming etching of MAX phases, such as KF, NaF, NH_4_F, and NH_4_HF_2_ [28].

HF etching and in situ HF-forming etching with HCl/LiF are common methods for MXene preparation, and their etching mechanisms of Al atomic layers in Ti_3_AlC_2_ are not the same. In situ HF-forming etching method does not corrode Al atoms in the internal region of Ti_3_AlC_2_ particles during initial etching because the etchant cannot approach that region, while HF etching method decomposes MXene polycrystals into single crystals along the grain boundaries, such that more Al edge positions are exposed to the etchant for reaction. As the reaction proceeds, the two methods etch similarly, with Ti_3_AlC_2_ being converted into Ti_3_C_2_T_x_. In fact, delamination of some parts of the layers initiates, and Al atomic layers inside particles are further etched. In other words, the etchant peels layer by layer along the vertical direction to the sheet base instead of etching each layer at a constant rate. The main difference in etching behaviors between the two methods is the etching ability at the Ti_3_AlC_2_ crystal grain boundaries. The etching strength of HF is greater than that of HCl/LiF and sufficient to penetrate the entire grain boundary to finally break the Ti_3_AlC_2_ polycrystalline particles into single-crystal particles. In contrast, crystalline particles remain intact even after 24 h of etching when using HCl/LiF [14].

### 2.3. HCl/HF Mixed Acid

Owing to the strong corrosivity of HF, high concentration and long etching time increase the defects in MXene layers and decreases the transverse dimensions, adversely affecting the electrical properties. Mild etching can be achieved by reducing the HF concentration or applying in situ HF-forming etching with HCl/LiF. Alternatively, a mixed acid can be used for mild etching. For instance, MAX phases can be etched with mixed acid of HCl and high-concentration HF [10,19,39], obtaining high productivity and high-quality MXenes with wide applicability. Mixing HCl, HF, and water in a volume ratio of 6:1:3 and then adding MAX powder to the mixture, etching can be performed with continuous stirring for 24 h at room temperature, and washing should be repeated after etching until the pH value is greater than 6. Subsequently, centrifugal sediments are added to LiCl solutions for stirring over at least 4 h for intercalation. Finally, the dispersion is centrifuged and washed until the upper liquid turns black, obtaining water dispersion of single or a few layers. Using this method, -Cl groups and more water molecules can be added to the surface of Ti_3_AlC_2_. In addition, the interlayer distance of MXene layers can be enlarged without ultrasonic delamination, thus minimizing adverse effects on the performance.

### 2.4. Additional Preparation Methods

In addition to the above several common and reliable methods, there is a large number of other innovative methods to choose from. They have been discovered and proposed in succession to achieve simpler etching conditions, diversified properties, and better performances.

NH_4_F hydrothermal method: Wang et al. [40] proposed the hydrothermal ammonium fluoride method, in which 5 g NH_4_F is dissolved into 60 mL deionized water by magnetic stirring and then 0.5 g Ti_3_AlC_2_ powder is added to the solution under intense stirring. The process continues by sealing the reaction mixture in an 80 mL polytetrafluoroethylene-lined high-pressure autoclave maintained at 150 °C and cooling to room temperature naturally. The Ti_3_AlC_2_ material prepared by this method can be used in high-performance supercapacitors.

Alkali etching: Li et al. [41] prepared Ti_3_C_2_T_x_ by an alkali-assisted hydrothermal method without element F during processing. Some oxide/hydroxide layers appear on the Ti_3_AlC_2_ surface, and even after using aqueous HF solution to remove them, subsequently etching Al forms new oxide/hydroxide layers of Al under exposure to alkaline conditions. Therefore, Ti_3_AlC_2_ powder should be treated by a hydrothermal reaction with aqueous NaOH solution of 27.5 mol·L^−1^ at 270 °C. This hydrothermal method is carried out in an argon atmosphere to mitigate sample oxidation, finally obtaining multilayer Ti_3_C_2_T_x_ with a purity of about 92 wt%. Although fluorine-free MXene can be produced using this method, the process is complicated.

Electrochemical etching: Yang et al. [42] applied fluorine-free electrochemical etching based on corrosion of a Ti_3_AlC_2_ anode in a binary aqueous electrolyte. Al is inserted in situ into ammonium hydroxide after dissolution, allowing the preparation of a Ti_3_C_2_T_x_ sheet with a maximum size of 18.6 μm, and the productivity of the resulting single-layer MXene can reach 90%.

Fluoride-containing molten salt etching: Urbankowski et al. [43] obtained Ti_4_N_3_T_x_ by using molten fluoride salts (e.g., KF, LiF, NaF) to etch Al from Ti_4_AlN_3_ powder in an argon atmosphere at 550 °C. Density functional theory confirmed that magnetism appears on Ti_4_N_3_T_x_ without surface groups and decreases significantly with these groups.

Lewis-acid molten-salt method: Li et al. [44] replaced element A in a MAX phase with late-transition metal halides in their proposed Lewis-acid molten-salt method. First, Al in the MAX phase (Ti_3_AlC_2_, Ti_2_AlC, Ti_2_AlN, and V_2_AlC) is replaced by Zn in melting ZnCl_2_, thereby producing a new MAX phase. Melting ZnCl_2_ shows strong Lewis acidity and plays the role of H^+^, while Cl^−^ plays the role of F^−^. MXene (e.g., Ti_3_C_2_Cl_2_, Ti_2_CCl_2_) can be peeled from a new MXene phase (e.g., Ti_3_ZnC_2_, Ti_2_ZnC) under excess ZnCl_2_. Etching by melting salts using Lewis acid provides a green and feasible approach for HF-free MXene preparation, and the electrochemical properties can be improved by containing -Cl groups.

## 3. Preparation of MXene/Elastomer Nanocomposites

### 3.1. Latex Blending

For latex blending, water dispersion or powder of MXene is mixed with latex and stirred to uniform dispersion. Then, a large amount of water is separated by filtration or flocculation, obtaining MXene/elastomer nanocomposites after drying. The superb hydrophilicity of MXene allows dispersion into latex using water as the solvent and interaction with the matrix through hydrogen bonds or electrostatic forces. Thus, uniform dispersion in the matrix, low filler content, and excellent comprehensive performance are achieved. Owing to the lamellar structure of MXene and the presence of -OH on the surface, lamellar stacking easily occurs after adding MXene. This problem can be mitigated and even suppressed by prior MXene surface modification or freeze-drying after blending, thereby improving the dispersion and compatibility of MXene in the matrix.

**Figure 4:**
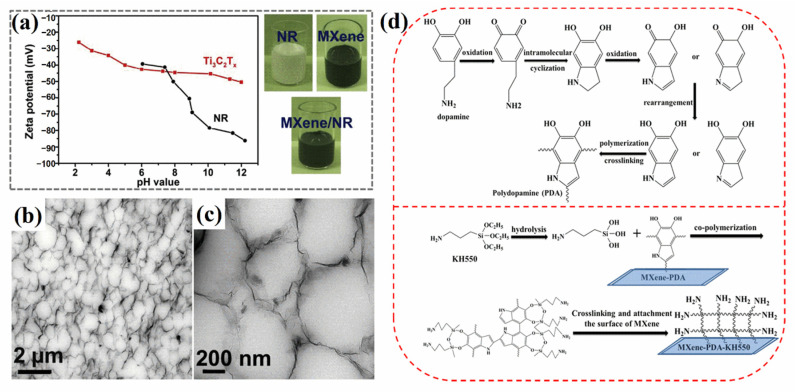
(**a**) Negative Zeta potentials of Ti_3_C_2_T_x_ suspensions and NR latex (Reprinted with permission from Ref. [20]. Copyright 2019, copyright Elsevier). (**b**,**c**) Transmission electron microscopy images of the network structure of MXene/NR (Reprinted with permission from Ref. [20]. Copyright 2019, copyright Elsevier). (**d**) Diagram of reaction of dopamine self-polymerization on surface of MXene and grafting of KH550 (Reprinted with permission from Ref. [46]. Copyright 2021, copyright Elsevier).

Luo et al. [20] mixed and stirred a Ti_3_C_2_T_x_ suspension with NR latex and prepared a Ti_3_C_2_T_x_/NR film by vacuum filtration to prepare a cross-linked Ti_3_C_2_T_x_/NR film with dicumyl peroxide as the cross-linking agent. The electrostatic repulsion between the negatively charged MXene and NR latex (Figure 4a shows that both MXene and NR have negative Zeta potentials) ensures that the MXene layer independently forms an interconnected 3D network structure between NR particles (Figure 4b,c), thus achieving excellent dispersion, and electrons can transfer efficiently and rapidly throughout the whole structure. This stable network endows nanocomposites with excellent electrical conductivity and extraordinary tensile properties. Yang et al. [45] also prepared MXene/NR composite films by vacuum filtration. Introducing NR provides hydrophobicity to nanocomposites. Hence, the oxidation of MXene can be inhibited by encapsulation in NR, preventing easy MXene oxidation in the presence of water.

Li et al. [47] dispersed Ti_3_C_2_T_x_ into styrene–butadiene rubber (SBR) latex and prepared Ti_3_C_2_T_x_/SBR composites by ordinary drying and freeze-drying. When the content of Ti_3_C_2_T_x_ was only two parts per hundred rubber (phr), the aggregation of Ti_3_C_2_T_x_ started to appear in the nanocomposites prepared by the ordinary drying method. Compared with ordinary drying method, freeze- drying method resulted in the dispersion of Ti_3_C_2_T_x_ thin sheets as monolayers in the nanocomposites with Ti_3_C_2_T_x_ of 2 and 4 phr. This is because rapid freezing of a Ti_3_C_2_T_x_/SBR aqueous solution limits the restacking between Ti_3_C_2_T_x_ sheets, owing to hydrogen bonding and van der Waals forces. Consequently, Ti_3_C_2_T_x_ can be uniformly dispersed in the matrix. Aakyiir et al. [48] mixed a Ti_3_C_2_T_x_ suspension with butadiene–acrylonitrile rubber (NBR) latex and prepared MXene/NBR composites through flocculation. Infrared spectroscopy revealed that hydrogen bonds formed instantaneously between -OH on the surface of Ti_3_C_2_T_x_ and unsaturated carbon–carbon double bonds of NBR after mixing the two materials, indicating that Ti_3_C_2_T_x_ can be well-dispersed in the matrix.

### 3.2. Solution Blending

For solution blending, an elastomer matrix is dissolved in an organic solvent, such as toluene, tetrahydrofuran, and N, N-dimethylformamide. Then, MXene in the solvent is ultrasonically dispersed and mixed with the matrix solution. Finally, MXene/elastomer nanocomposites are prepared by flocculation or casting. This method is simple and easy to perform, being suitable for some elastomers without latex. Lu et al. [49] adopted the solution blending method to prepare MXene/ethylene propylene diene monomer rubber (EPDM) composites using toluene as the solvent. This composite exhibited a low percolation threshold of 2.7 wt%. For a MXene content of 6%, the electrical conductivity of MXene/EPDM reached 106 S·m^−1^, the EMI shielding effectiveness (SE) in the X-band reached 48 dB, and the thermal conductivity reached 1.57 W·m^−1^·K^−1^.

Many polar groups on the surface of MXene result in poor dispersion in weak polar or non-polar solvents. Consequently, composites prepared by solution blending show low performance. MXene is usually modified by approaches such as adding silane coupling agents, polyphenols, quaternary ammonium salts, surfactants, etc. [50], to enhance compatibility in solvents and elastomer matrices as well as dispersion. Qu et al. [46] modified polydopamine (PDA) on the surface of MXene nanosheets and then grafted KH550 to obtain MXene-PDA-KH550 (Figure 4d). Then, they prepared NBR-modified MXene composites by mixing NBR with modified MXene by solution blending, using tetrahydrofuran as the solvent. After modification with PDA and KH550, MXene was more easily combined with the NBR segment owing to the -NH_2_ group on the surface, thus improving compatibility. Segment motion consumed more energy owing to the increase in internal friction between the modified MXene and NBR segments, and the loss factor (tan*δ*) increased to approximately 1.0, leading to an effective damping temperature range of approximately 40 °C, which was higher than that of 25 °C for NBR, indicating good damping performance.

### 3.3. Backfill Matrix Method

For the backfill matrix method, a 3D MXene structure should be first constructed to form a conductive network. The network is then immersed in latex or a solution of elastomer, or the latex/solution is directly cast into a mold with the 3D MXene network to backfill the elastomer. The MXene/elastomer nanocomposite with a 3D network is prepared by drying, curing, and other steps. Owing to the good electrical conductivity and porosity of the prebuilt 3D MXene network, MXene/elastomer nanocomposites prepared by the backfill matrix method are commonly applied to EMI shielding, sensing, and other areas.

MXene dispersions can be prepared as light and low-density aerogels by freeze-drying and other methods, but the gelling ability of MXene is weak. Thus, graphene oxide [51], polyvinyl alcohol [52], alginate, or other solutions mixed with MXene are usually required to prepare porous aerogels to form a stable network through hydrogen bonding and electrostatic, gel, and ionic interactions [53]. Alternatively, MXene can be directly adsorbed on skeletons, such as cellulose scaffolds or nickel foams, to form a 3D network. Finally, the porous framework is impregnated into elastomers or latexes (e.g., PDMS) to increase the stability and durability of the structure.

### 3.4. Additional Methods

MXene/elastomer nanocomposites can also be prepared by methods such as mechanical blending, melt blending [54], spin coating [55], and dip coating [56,57]. Mechanical blending is the most common blending method used to prepare composite materials. However, given the mechanical flexibility of MXene, the sheets are easily stacked through hydrogen bonds after drying. Compared with other nanofillers, MXene hardly blends with elastomers mechanically. Moreover, the intense heat generated by mechanical shearing force or heating conditions oxidizes MXene sheets.

## 4. Properties and Applications of MXene/Elastomer Nanocomposites

### 4.1. Mechanical Properties

Elastomer, with its high elasticity, is a very important flexible material that can provide buffer protection, adaptability to deformation, long-term stability, and other advantages. Its excellent mechanical properties are indispensable for various applications. Nanofillers reinforce elastomers while functionalizing elastomers. The reinforcing effect of MXene on elastomers is not inferior to that of graphene, which is also a 2D layered material, and may even achieve higher performance. Similar to the functionalization of elastomers, the effect of MXene on the mechanical properties of elastomers has attracted much attention.

**Figure 5:**
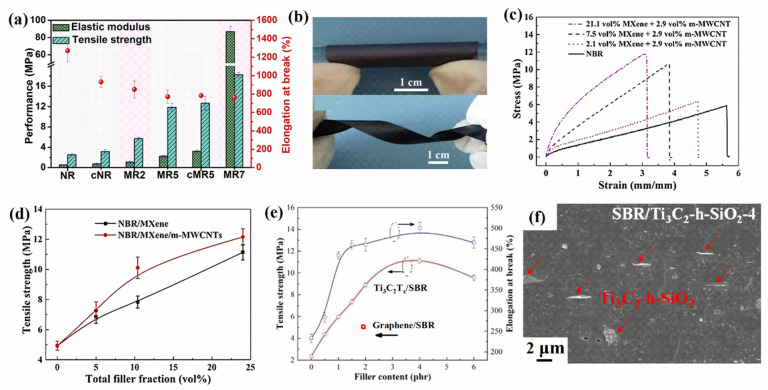
(**a**) Tensile strengths and elastic moduli of MXene/NR composite films (Reprinted with permission from Ref. [20]. Copyright 2019, copyright Elsevier). (**b**) MXene/NR film twisted up to 360° (Reprinted with permission from Ref. [45]. Copyright 2020, copyright Elsevier). (**c**) Stress–strain curves for MXene/CNT/NBR nanocomposites (Reprinted with permission from Ref. [59]. Copyright 2021, copyright Elsevier). (**d**) Tensile strengths of NBR/MXene and NBR/MXene/CNT (Reprinted with permission from Ref. [59]. Copyright 2021, copyright Elsevier). (**e**) Tensile strength and elongation at break of Ti_3_C_2_T_x_/SBR (Reprinted with permission from Ref. [47]. Copyright 2019, copyright RSC). (**f**) Scanning electron microscopy image of SBR/Ti_3_C_2_T_x_-h-SiO_2_ (Reprinted with permission from Ref. [60]. Copyright 2021, copyright Elsevier). (cNR: cross-linked NR film; MR2: Ti_3_C_2_T_x_/NR film with 1.18 vol% Ti_3_C_2_T_x_; MR5: Ti_3_C_2_T_x_/NR film with 3.10 vol% Ti_3_C_2_T_x_; cMR5: cross-linked Ti_3_C_2_T_x_/NR film with 3.10 vol% Ti_3_C_2_T_x_; MR7: Ti_3_C_2_T_x_/NR film with 6.71 vol% Ti_3_C_2_T_x_.)

Luo et al. [20] prepared Ti_3_C_2_T_x_/NR composite films by latex blending. The sturdy and stable 3D MXene network and electrostatic interaction significantly enhanced the mechanical properties of NR. When the content of MXene was 6.71 vol%, the tensile strength and modulus of the Ti_3_C_2_T_x_/NR composite films increased by 7 and 15 times, respectively, compared with NR films (Figure 5a). The addition of MXene nanosheets enhances the flexibility and elasticity of NR molecular segments. Yang et al. [45] enhanced the strength and ductility of MXene films by introducing NR. The tensile strength of MXene/NR films increased from 23 MPa for MXene to 53 MPa for 40 wt% NR, and the elongation at break increased from 0.9% to 4.5% for 60 wt% NR. The MXene/NR composite film could be easily wrapped around a glass rod over 360° (Figure 5b), and the number of folding cycles could reach 7425, significantly exceeding the 119 cycles of a MXene film, indicating excellent foldability. The interconnected layered structure of MXene/NR composite films facilitated the efficient transfer of stress from the flexible NR macromolecular chains to the rigid MXene nanosheets, enhancing the tensile strength of the films. Sheng et al. [58] modified MXene with polyethylene glycol at a weight ratio of 1:2 and prepared polyethylene glycol–MXene/TPU by melt blending with TPU. Compared with TPU, when the content of polyethylene glycol–MXene was 0.5 wt%, the tensile strength of polyethylene glycol–MXene/TPU nanocomposites increased from 14.0 to 20.6 MPa, and the elongation at break increased from 1580% to 1857%.

Aakyiir et al. [48] prepared MXene/NBR composites by latex blending. The tensile strengths of the composites increased from 11 to 21 MPa (7.5 vol% MXene), and the elongation at break increased from 500% to 640% (3.9 vol% MXene). In addition, the Young’s moduli increased from 2 to 13 MPa (14.0 vol% MXene). The significantly enhanced tensile strengths and Young’s moduli of the composites were attributed to the monolayer MXene materials having strengths and moduli comparable to those of graphene oxide and molybdenum disulfide. Furthermore, the surface groups of MXene can form hydrogen bonds and other forces with the NBR macromolecules. Aakyiir et al. [59] also prepared MXene/CNT/NBR composites after electrostatic assembly of cetyltrimethylammonium bromide (CTAB)-modified multi-walled CNTs (fixed amount of 2.9 vol%) with MXene. The surface modification increased the compatibility between nanofillers and NBR and promoted the interaction between hybrid fillers and elastomeric macromolecules, resulting in nanocomposites containing MXene/CNT hybrid fillers and achieving significantly higher performance than the same NBR composites under MXene loading (Figure 5c,d).

Li et al. [47] dispersed Ti_3_C_2_T_x_ into styrene–butadiene latex and then freeze-dried the solution to prepare Ti_3_C_2_T_x_/SBR composites. For a given filler fraction, the tensile strengths of the Ti_3_C_2_T_x_/SBR composites were higher than those of graphene/SBR composites (Figure 5e), indicating that reinforcing Ti_3_C_2_T_x_ is considerably better than reinforcing the same 2D layered materials of graphene. Ma et al. [60] fabricated SBR/Ti_3_C_2_T_x_-h-SiO_2_ elastomer composites by electrostatically assembling KH550-modified SiO_2_ with MXene and mixing with styrene–butadiene latex. The surface nanoprotuberances of KH550-modified SiO_2_ enabled more rubber segments to be captured and immobilized, thereby enhancing the interfacial interactions. This hybridization solved the agglomeration of MXene and led to uniform dispersion in the matrix (Figure 5f). The tensile strength and elongation at break of SBR/Ti_3_C_2_T_x_-h-SiO_2_ were always higher than those of SBR and SBR/Ti_3_C_2_T_x_, and the tensile strength of SBR/Ti_3_C_2_-h-SiO_2_ with a 4 phr filler increased from 4.09 and 7.52 MPa to 11.21 MPa. Jia et al. [61] solved the stacking problem of MXene sheets by using tetraethyl orthosilicate to grow SiO_2_ on the surface of Ti_3_C_2_T_x_ in situ and mixed it with styrene–butadiene latex to prepare a uniformly dispersed SBR/Ti_3_C_2_T_x_-g-SiO_2_ composite. The tensile strength of SBR/Ti_3_C_2_T_x_ with 6 phr filler only increased from 4.09 to 9.05 MPa compared with NBR, and the elongation at break increased from 271% to 461%, while the tensile strength of SBR/Ti_3_C_2_T_x_-g-SiO_2_ with 4 phr filler significantly increased to 14.1 MPa, and the elongation at break increased to 522%.

### 4.2. EMI Shielding

Conductive elastomer nanocomposites are being increasingly used in EMI shielding owing to their flexibility, light weight, corrosion resistance, and excellent processability. Compared with carbon nanomaterials, the layered structure, abundant surface groups, excellent electrical conductivity, and hydrophilicity of MXene can improve EMI shielding using elastomer materials. In addition, MXene/elastomer nanocomposites are suitable for flexible and retractable electronics.

**Figure 6:**
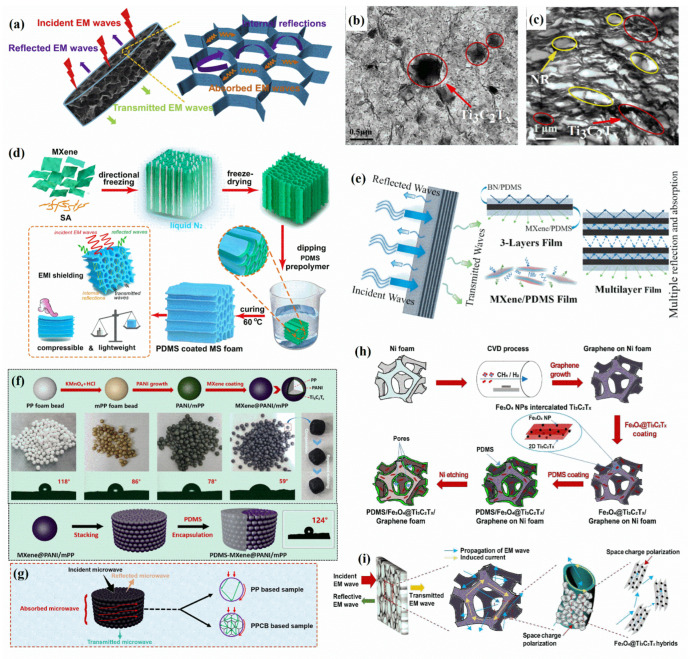
(**a**) EMI shielding mechanisms of MXene/NR nanocomposite films (Reprinted with permission from Ref. [20]. Copyright 2019, copyright Elsevier). Transmission electron microscopy images of (**b**) brick–mortar and (**c**) honeycomb structures (Reprinted with permission from Ref. [62]. Copyright 2021, copyright Elsevier). (**d**) Schematic of fabrication of MXene/sodium alginate hybrid aerogel and PDMS-coated foam (Reprinted with permission from Ref. [16]. Copyright 2019, copyright Elsevier). (**e**) Schematic of EMI shielding mechanism of MXene/BN/PDMS multilayer films (Reprinted with permission from Ref. [55]. Copyright 2020, copyright Elsevier). (**f**) Schematic of fabrication of PDMS/MXene@PANI/PP composite foams (Reprinted with permission from Ref. [63]. Copyright 2020, copyright Elsevier). (**g**) EMI shielding mechanisms of composite foams (Reprinted with permission from Ref. [63]. Copyright 2020, copyright Elsevier). (**h**) Schematic of fabrication of Fe_3_O_4_@Ti_3_C_2_T_x_/graphene/PDMS (Reprinted with permission from Ref. [64]. Copyright 2020, copyright Elsevier). (**i**) Schematic of electromagnetic wave absorption in Fe_3_O_4_@Ti_3_C_2_T_x_/graphene/PDMS composite (Reprinted with permission from Ref. [64]. Copyright 2020, copyright Elsevier). (SA: sodium alginate; EM: electromagnetic; MS: MXene/sodium alginate; PPCB: polypropylene/carbon black).

Luo et al. [20] prepared Ti_3_C_2_T_x_/NR composites with 0.58–6.71 vol% MXene content by latex blending. The MXene flakes were uniformly distributed in the NR matrix owing to the electrostatic repulsion between the negative charges of MXene and NR, forming an interconnected filling network. The resulting Ti_3_C_2_T_x_/NR nanocomposite film (with a thickness of 246 ± 5 μm) containing 6.71 vol% MXene achieved a high electrical conductivity of 1400 S·m^−1^ and an EMI SE of 53.6 dB. The porous network composed of MXene transmits incident electromagnetic waves on the porous surface and interface, and the waves can be dissipated and attenuated by multiple scattering and interface polarization, such that the absorption loss (SE_A_) is significantly greater than the reflection loss (SE_R_) (Figure 6a). Wang et al. [62] prepared Ti_3_C_2_T_x_/NR-composite-film membranes by latex blending. The composite films with brick–mortar structures (Figure 6b) were transformed into porous honeycomb structures (Figure 6c) after vulcanization. During crosslinking, the high viscosity of NR severely restricted the free diffusion of Ti_3_C_2_T_x_ nanosheets, resulting in Ti_3_C_2_T_x_ accumulation at the edge of the NR region, finally forming a tightly packed honeycomb structure. Compared with the randomly distributed brick–mortar structure, the Ti_3_C_2_T_x_ flakes established a continuous conductive network after vulcanization, and the porous conductive surface dissipated electromagnetic waves more effectively. An unvulcanized NR/Ti_3_C_2_T_x_ (1:10) composite film achieved an SE of 57.1 dB. After vulcanization to form a more regular honeycomb structure, the SE increased to 63.5 dB, and the behaviors of the two composite films were mainly based on the absorption loss mechanism.

The results of electrostatic repulsion and other interactions between natural latex and MXene ensure the even dispersion of MXene in the matrix and form an ordered network, while MXene nanocomposites prepared from other elastomer latexes are difficult to surpass in performance, such as the nanocomposite prepared by mixing nitrile latex with MXene, which has an SE of 49 dB when the MXene content is 19.6 vol% [65]. In addition, the filler content is significantly higher than that of NR. Therefore, more scholars currently focus on the construction of 3D conductive networks, the design of porous and multilayer structures, or the synergistic hybridization of various nanofillers in advance to enhance the EMI shielding performance of elastomer nanocomposites.

Three-dimensional networks and multilayer structures play significant roles in the absorption of electromagnetic waves, which are suppressed after multiple reflections and scattering in these structures, increasing SE_A_ and reducing the proportion of SE_R_, thus preventing secondary pollution of electromagnetic waves. Wu et al. [16] fabricated MXene/sodium alginate/PDMS foam (Figure 6d) by constructing MXene/sodium alginate aerogel with a directional arrangement and then vacuum backfilling PDMS. With increasing MXene content, the EMI SE and SE_A_ of MXene/sodium alginate aerogel were enhanced, but the SE_R_ remained below 5 dB, indicating that it mainly weakened electromagnetic waves through absorption. After dipping into PDMS to form a coating, the SE of foam containing 74.07 wt% MXene was 53.9 dB, and after 500 compression/release cycles at 30% strain, the SE still reached 48.2 dB. Such an excellent performance indicates that PDMS coating strengthens the network structure to withstand compressive loads. Liu et al. [55] prepared MXene/BN/PDMS nanocomposites with an ordered layered structure by alternately spin coating BN/PDMS insulating/thermally conductive layers with MXene/PDMS (10 wt% MXene) conductive layers. With an increasing number of layers, the SE_R_ changed less, and the SE_A_ increased significantly. For a nanocomposite film with 11 layers, the SE reached 35.2 dB. After the incident electromagnetic wave directly penetrated the BN layer, part of the electromagnetic wave was immediately reflected when it encountered the MXene layer, and the rest of the electromagnetic wave entered the multilayer film, being further attenuated by multiple reflections and absorptions between conductive layers (Figure 6e). Jia et al. [63] prepared MXene@polyaniline/polypropylene foam (MXene@PANI/PP), which PP foam microbeads treated by oxidation, PANI and MXene were arranged and stacked in a mold cylindrical, and sequentially immersed MXene@PANI/PP in PDMS (Figure 6f). When the MXene content reached approximately 0.0449 vol%, the SE of the PDMS/MXene@PANI/PP with a thickness of 12 mm increased from approximately 8 to 39.8 dB. Hence, the conductive interface generated between adjacent foam beads enhanced the internal 3D network, resulting in multiple reflections and absorptions of electromagnetic waves between the interfaces inside the material, indicating the importance of a 3D conductive network constructed by encapsulation (Figure 6g). Jia et al. [66] mixed CTAB-modified Ti_3_C_2_T_x_ with shape-memory elastomer 1, 4-trans polyisoprene by solution blending and immersed the compressible carbon foam backbone in a solution for coating the surface. The SE of 1, 4-trans polyisoprene/carbon foam with a thickness of about 10 mm in the X-band reached 25.3 dB, and, after adding CTAB-MXene, the SE of the composite containing 20 wt% CTAB-MXene increased to 44.7 dB. This composite material exhibited excellent thermal/electrically stimulated shape-memory behavior that allowed tuning of the EMI shielding properties under self-fixing mechanical deformation.

The synergistic effect of the hybridization of various nanofillers can enhance the EMI shielding of composites and even simultaneously generate conductive surface impedance mismatch, interface polarization, and magnetic loss. Nguyen et al. [64] developed a lightweight and flexible 3D porous Fe_3_O_4_@Ti_3_C_2_T_x_/graphene/PDMS composite (Figure 6h,i). First, graphene was synthesized in nickel foam by chemical vapor deposition to form a tightly interconnected conductive network, and Fe_3_O_4_ was inserted into the nanosheets of Ti_3_C_2_T_x_. Then, the nickel foam was immersed in the Ti_3_C_2_T_x_ dispersion, dried, and immersed in PDMS again. The nickel foam was etched to obtain a hybrid nanocomposite. After adding an Fe_3_O_4_@Ti_3_C_2_T_x_ hybrid filler, the average SE values in the X-band and Ka-band were 80 and 77 dB, respectively. At 8.7 GHz, the values reached 83.6 dB, and at 39.6 GHz they reached 78.9 dB, with the SE_A_ accounting for more than 80%. Song et al. [67] prepared a flexible stretchable elastomer with a 3D conductive network by chemically depositing in-house Fe_3_O_4_@Ti_3_C_2_T_x_ nanosheets mixed with 3, 4-dihydroxyphenylacetic acid-modified epoxidized NR latex. The Fe_3_O_4_@Ti_3_C_2_T_x_ nanosheets were formed into a 3D isolated conductive network by the excluded volume effect of latex. The high electrical conductivity of Ti_3_C_2_T_x_ and the magnetic properties of Fe_3_O_4_ nanoparticles provided an EMI SE of the elastomer up to 58 dB in the X-band, and it remained stable after repeated deformation.

### 4.3. Flexible Sensors

In applications such as human motion detection, health monitoring, and human–computer interaction, the demand for flexible sensors for portable and wearable electronic devices is increasing. MXene has attracted widespread attention in the design and preparation of flexible sensors owing to the excellent conductivity and modifiable terminated functional groups on its surface. In addition, a flexible elastomer matrix is indispensable for flexible sensors.

**Figure 7:**
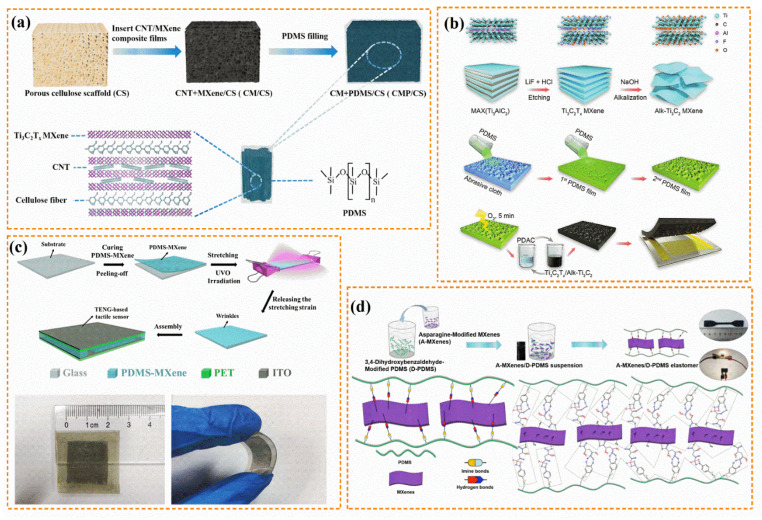
(**a**) Schematic of synthetic procedure for multifunctional elastic CNT/Ti_3_C_2_T_x_/PDMS/CS composites. Compressibility of composites for 1000 cycles at 30% strain (Reprinted with permission from Ref. [68]. Copyright 2021, copyright Elsevier). (**b**) Schematic of fabrication of Alk-Ti_3_C_2_ and PDMS films with positive structures and flexible pressure sensors (Reprinted with permission from Ref. [29]. Copyright 2021, copyright Wiley-VCH). (**c**) Schematic of fabrication and real images of triboelectric tactile sensor based on wrinkled PDMS/MXene composite films (Reprinted with permission from Ref. [70]. Copyright 2020, copyright Elsevier). (**d**) Main preparation steps of A-MXene/D-PDMS elastomer and interaction between A-MXene and D-PDMS (Reprinted with permission from Ref. [71]. Copyright 2020, copyright ACS). (PDAC: poly(diallyl dimethylammonium chloride); PET: polyethylene terephthalate; ITO: indium tin oxide).

As the most common matrix, PDMS provides sensors with flexibility, structural stability, and durability. The backfilling matrix method is a method to prepare MXene/PDMS flexible sensors. First, a 3D MXene conductive network is constructed to dip it into PDMS and cure it to obtain MXene/PDMS sensors with high sensitivity and cyclic stability. Wang et al. [68] prepared a CNT/Ti_3_C_2_T_x_/PDMS/cellulose-scaffold flexible piezoresistive sensor (Figure 7a) based on a multichannel 3D cellulose scaffold, dispersion of polyvinylpyrrolidone-modified CNTs electrostatically assembled with Ti_3_C_2_T_x_, and PDMS, using the abovementioned method. The flexible sensor achieved compressibility, structural stability, and excellent fatigue resistance. Moreover, the sensor had approximately the same current variation after 1000 cycles at 30% strain, indicating its excellent cycling stability and durability. In addition, the sensor had a gauge factor of 3.94, indicating a high sensitivity between the compressible strain and the output current. Song et al. [69] used nickel foam as a 3D sacrifice template to fabricate a hollow-structured MXene/PDMS piezoresistive pressure sensor. During deformation, MXene nanosheets were in contact to form a dense conductive network. Therefore, the bendable sensor had a working range with bending angles ranging from 0 to 180°, a high Δ*R*/*R*_0_ of 90%, a broad working frequency range, and stability over 1000 cycles. Furthermore, some MXene/elastomer nanocomposites for EMI shielding with stable network structures and compressive permanent deformation have been used as flexible sensors through application of the backfilling matrix method, achieving multifunctionality [16,64].

Figure 7b,c show the preparations of the different flexible sensors with rough PDMS substrates. The wrinkles or convex structures of substrates help to increase the sensitivities and signal intensities of flexible sensors. Xiang et al. [29] produced a flexible piezoresistive sensor with NaOH-alkalized 3D crinkled Ti_3_C_2_ MXene (Alk-Ti_3_C_2_) conductive material and rough PDMS substrate. The flexible sensor was assembled with convex-structured PDMS coated with Alk-Ti_3_C_2_ and poly(diallyl dimethylammonium chloride) using the layer-by-layer method. In addition to PDMS with microstructures, crinkled Alk-Ti_3_C_2_ can further improve the sensitivity of the sensor. A flexible piezoresistive sensor achieved an ultrawide pressure range of 0–800 kPa, low detection limit of 17 Pa, ultrahigh sensitivity of 95.26–1104.38 kPa^−1^, fast response time of 100 ms, and excellent cycle stability of 3000 cycles at 300 kPa. This sensor is suitable for applications requiring multiple pressure measurements, such as wrist pulse, vocal-cord vibration, and weight measurements. Cai et al. [70] designed a flexible ultrasensitive triboelectric tactile sensor based on wrinkled PDMS/MXene composite films. The films were prepared by stretching and ultraviolet ozone irradiation treatment because a triboelectric nanogenerator (TENG) consists of a wrinkled PDMS/MXene film and two polyethylene terephthalate films. The triboelectric tactile sensor achieved ultrahigh sensitivities of 0.18 and 0.06 V·Pa^−1^ in ranges of 10–80 Pa and 80–800 Pa, respectively. The sensor can be applied to monitor complex human physiological signals, and it can be constructed as electronic skin to imitate the human tactile sensation.

MXene/elastomer nanocomposites with favorable dispersions of MXene also have excellent sensing properties via latex compounding for devices such as the MXene/SBR-based flexible sensor [47]. However, some elastomers lack latex with water as the solvent or high fluidity, such as PDMS. Therefore, the preparation of other elastomer-based flexible sensors usually consists of constructing an elastomer skeleton and then dipping the skeleton in MXene dispersion. Jia et al. [56] fabricated a flexible TPU/polyacrylonitrile mat through electrospinning and prepared a stretchable MXene/TPU/polyacrylonitrile flexible strain sensor via dip coating. The compatibility between MXene nanosheets and the TPU substrate was increased by introducing polyacrylonitrile into the flexible substrate, resulting in MXene nanosheets wrapped around TPU/polyacrylonitrile nanofibers. The MXene/TPU/polyacrylonitrile strain sensor was endowed with a wide sensing range of 0%–80%, fast response below 140.6 ms, low detection limit below 0.1%, excellent coating adhesion, and excellent durability surpassing 1750 cycles. Wang et al. [57] fabricated a CNT/TPU nanocomposite foam by salt templating and then dipped it in a dispersion of MXene and dried it. This high-performance foam-shaped TPU/CNT@MXene sensor showed a broad strain sensing range close to 100%, high sensitivity of gauge factor up to 363, and high cyclic stability. Furthermore, the composite foam achieved excellent gas permeability and an elastic modulus similar to those of skin, suggesting its comfort if used as a wearable sensor.

Flexible sensors are prone to scratches and cracks during continuous deformation, resulting in performance degradation. Therefore, the development of self-healing flexible sensors has been actively pursued. Owing to the functional groups on the surface, MXene is significantly more modifiable than nanomaterials such as graphene. This property and the interactions between biomolecules have increased attention toward modifying MXene and elastomers using biomolecules. Guo et al. [72] produced a self-healable supramolecular MXene/epoxidized NR sensor with serine-modified MXene and serine-modified epoxidized NR via latex compounding. The hydrogen-bonding interface between molecular chains allowed the elastomer to fully recover its initial mechanical and electrical properties at room temperature after complete fracture. With a high gauge factor of 107.43, low strain detection limit of 0.1%, fast response time of 50 ms, and excellent repeatability, a protein-inspired self-healable sensor accurately detected weak physiological activity, moisture changes in the human body, and even small changes after it was cut and healed. Similarly, Zhang et al. [71] designed a flexible stretchable self-healing sensor with MXene and amino-PDMS. Specifically, MXene and an amino-PDMS substrate were modified by aspartic acid and 3, 4-dihydroxybenzaldehyde via esterification and Schiff-base reactions, respectively (Figure 7d). The modified MXene nanosheets were well-dispersed in the amino-PDMS substrate, and the sensor showed excellent tensile properties, efficient self-healing, and high sensing performance.

### 4.4. Energy Storage and Conversion

Owing to its layered structure, abundant surface functional groups, metal conductivity, high negative zeta potential, large specific surface area, and good electrochemical properties, MXene can be used to construct advanced energy-storage and conversion devices exhibiting high energy and power densities, such as electrostatic capacitors, supercapacitors, and TENGs.

**Figure 8:**
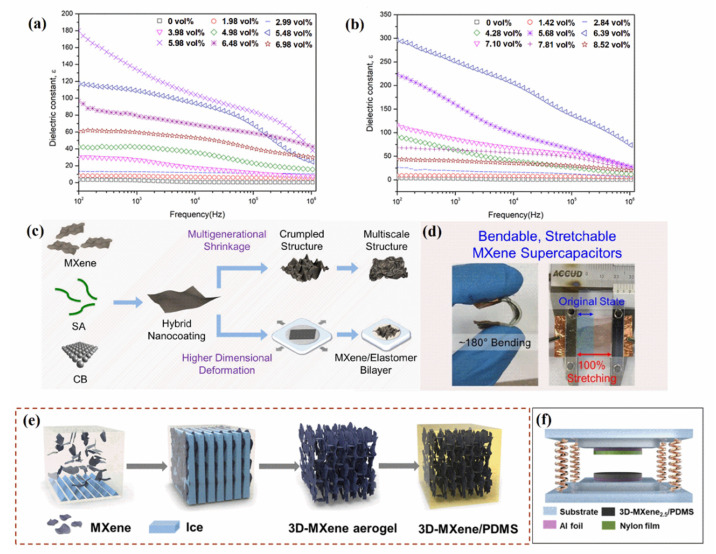
Dielectric constant of (**a**) Ti_3_C_2_T_x_/AE and (**b**) PANI-Ti_3_C_2_T_x_/AE (Reprinted with permission from Ref. [76]. Copyright 2021, copyright Elsevier). (**c**) Schematic of fabrication of high-areal-capacitance electrodes and highly bendable and stretchable supercapacitors (Reprinted with permission from Ref. [77]. Copyright 2018, copyright ACS). (**d**) The MXene/elastomer electrodes for bendable, stretchable supercapacitors (Reprinted with permission from Ref. [77]. Copyright 2018, copyright ACS). (**e**) Schematic of fabrication of 3D-MXene/PDMS (Reprinted with permission from Ref. [78]. Copyright 2019, copyright Elsevier). (**f**) Schematic of 3D-MXene/PDMS-based TENG (Reprinted with permission from Ref. [78]. Copyright 2019, copyright Elsevier). (SA: sodium alginate; CB: carbon black nanoparticle).

Elastomers with high flexibility and elasticity achieve high breakdown strength, but their dielectric constants are significantly lower than those of ceramics, limiting energy storage density. To develop modern electronics and power systems, dielectric elastomers with excellent dielectric constants and low dielectric loss factors are needed to build electrostatic capacitors with high energy densities that are lightweight and flexible and have high breakdown strengths. The percolation theory indicates that conductive fillers can be added to elastomers to improve the dielectric properties [73]. Jena et al. [74] prepared nanocomposites with Ti_3_C_2_T_x_ and ethylene–vinyl acetate (EVA) via solution blending. The composites containing 8 wt% Ti_3_C_2_T_x_ had higher dielectric constants and lower loss factors. The dielectric constant was 1.2 × 10^4^ at 25 °C, which was four orders of magnitude higher than that of EVA. The dielectric loss factor was 0.15 at 1 kHz, which was lower than that of composites containing 10 wt% and 12 wt% Ti_3_C_2_T_x_. The AC conductivity of the nanocomposite was 10^−8^ Ω^−1^·cm^−1^, being 25 times higher than that of EVA. Wei et al. [75] fabricated a MXene/PDMS bimodal-network percolation composite. First, terminal amino groups produced by the hydrolysis and condensation of hyperbranched polysiloxane were inserted into Ti_3_C_2_T_x_ nanosheet layers to expand the interlayer spacing. Then, short-chain PDMS was modified with Ti_3_C_2_T_x_ to further improve dispersion and compatibility. Finally, the modified MXene was mixed and cross-linked with long-chain PDMS. The dielectric constant of hyperbranched polysiloxane-Ti_3_C_2_T_x_/PDMS achieved was 23.7 at a low percolation threshold of 1.43 vol%, which was 1.5 and 8.5 times higher than that of Ti_3_C_2_T_x_/PDMS and PDMS, respectively. The loss factor (0.11) was also relatively low at 1 kHz. Gao et al. [76] mixed PANI in situ-modified MXene with acrylic resin elastomer (AE). The dielectric constant of the AE nanocomposite with 5.98 vol% Ti_3_C_2_T_x_ was 178 and that with 6.39 vol% PANI-Ti_3_C_2_T_x_ was 298 (85 times that of AE) at 100 Hz (Figure 8a,b). Moreover, the loss factors of the Ti_3_C_2_T_x_/AE and PANI-Ti_3_C_2_T_x_/AE composites were only 0.55 and 0.29, respectively.

Supercapacitors are energy-storage devices with characteristics between traditional capacitors and batteries. They can charge and discharge quickly and reversibly, and their power densities and cycle lives are considerably better than those of batteries. MXene/elastomer-based electrodes, as constituent elements of supercapacitors, allow the achievement of desirable electrochemical and mechanical properties. Chang et al. [77] demonstrated a stretchable, bendable, and efficient MXene-based supercapacitor (Figure 8c,d). MXene/elastomer-based electrodes with 225% strain were prepared by transferring crumpled multiscale-structured MXene nanocoatings onto elastomeric substrates. These electrodes exhibited high electrochemical performances of 395, 390, and 362 F·cm^−3^ at 0%, 50%, and 80% strains, respectively. Stretchable MXene-based and activated carbon electrodes formed a stretchable asymmetric supercapacitor with 180° bendability and 100% stretchability. This stretchable supercapacitor exhibited an effective energy density of 5.5 Wh·kg^−1^ in an Li_2_SO_4_–polyvinyl alcohol gel electrolyte and an effective energy density of 4.9 Wh·kg^−1^ after 100% deformation.

TENGs can convert mechanical energy, light energy, and other environmental excitations into electrical energy for flexible, scalable electronics. The MXene/elastomer flexible friction material is the most important part of TENGs and serves as a contact electrode to enhance the energy-conversion efficiency of TENGs by modulating electronegativity, conductivity, and dielectric properties. Wang et al. [78] fabricated a 3D-MXene/PDMS composite as a negative friction material for TENG by impregnating MXene aerogel in PDMS. Then, they designed a TENG with contact-separation mode by using a substrate and spring (Figure 8e,f). At an impact frequency of 2 Hz, the output voltage of TENG reached 45 V, higher than that of a TENG based on PDMS (33 V). In addition, the high output current of their TENG reached 0.6 μA, this being approximately three times that of a TENG based on PDMS. The improvement in output performance was attributed to the introduction of MXene into the PDMS dielectric to form microcapacitors. Thus, the dielectric constant improved, resulting in the higher capacitance and surface charge density of the nanocomposites. The dielectric constant was above 100 in the frequency range of 1 kHz–1 MHz, significantly higher than that of PDMS. Jiang et al. [79] produced a MXene/PDMS porous film electrode via simple blending and spin coating and then designed a TENG based on MXene porous film integrated with a laser-induced graphene electrode. This TENG achieved a maximum output voltage around 119 V and a maximum output current of approximately 11 μA. Liu et al. [30] prepared a stretchable TENG by using MXene/PDMS as a friction electrode. This TENG could simultaneously convert mechanical and light energy into electrical energy. The highly electronegative surface and intense surface-plasmon-excitation property of MXene enabled an output voltage and electric current of up to 453 V and 131 μA, respectively.

### 4.5. Flame Retardants

MXene has also been applied in flame retardancy, smoke suppression, and fire prevention, primarily owing to the layered blocking effect of 2D nanosheets and the MXene catalysis promoting the formation of carbon protective films. Modification and hybridization currently constitute the most suitable strategies for improving the dispersion of MXene nanosheets in a matrix and obtaining synergistic flame retardants.

**Figure 9:**
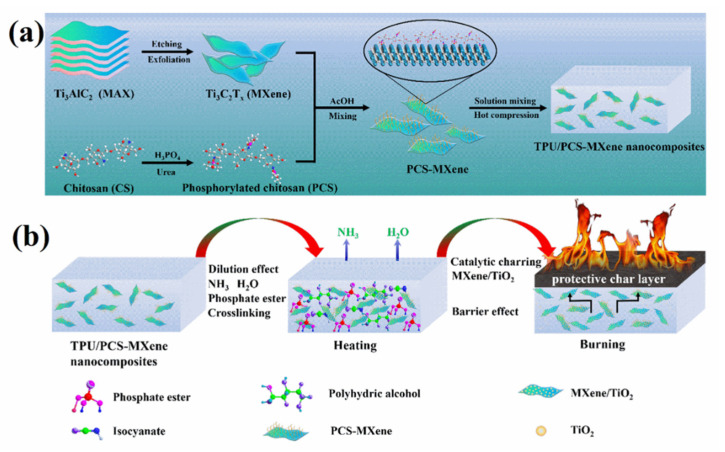
(**a**) Illustration of the preparation and (**b**) possible flame-retardant mechanism of TPU/PCS-MXene nanocomposites (Reprinted with permission from Ref. [80]. Copyright 2022, copyright Elsevier).

Yu et al. [31] prepared CTAB and tetrabutyl phosphine chloride-modified Ti_3_C_2_T_x_ MXene nanosheets and then blended the two modified MXenes with TPU. Compared with TPU only, the flame-retardant and smoke-suppression properties of composites with only 2 wt% of modified MXenes were significantly improved, likely owing to the good dispersion, catalysis, and barrier of MXene nanosheets in TPU. In cone calorimeter tests, the peak heat-release rates were reduced by 51.2% and 52.2% and the peak smoke-production rates were reduced by 57.1% and 57.4%, while peak CO production was reduced by 39.4% and 41.6% and peak CO_2_ production was reduced by 49.7% and 51.7% after adding the two modified MXenes, respectively. Luo et al. [80] obtained a phosphorylated chitosan (PCS) suspension by stirring and reacting chitosan and urea dispersed in phosphoric acid. They then produced PCS-MXene by adding and reacting the suspension dropwise to a MXene dispersion and finally mixing PCS-MXene with TPU by solution blending (Figure 9a). The excellent dispersion of PCS-MXene and the hydrogen bonding between PCS-MXene and the matrix prevented molecular-chain breakage, thereby enhancing the tensile properties of the composites by about 10 MPa compared with TPU only. TPU is an extremely flammable polymer with a peak heat-release rate and total heat release as high as 910.7 kW·m^2^ and 67.1 MJ·m^2^, respectively. The addition of 3 wt% MXene reduced the peak heat-release rate and total heat release of MXene/TPU to 486.7 kW·m^2^ and 56.0 MJ·m^2^, respectively. However, after filling with 3 wt% PCS-MXene, these values for the composites were reduced to 303.6 kW·m^2^ and 53.0 MJ·m^2^, respectively, which values were similar to those obtained after the introduction of 3 wt% PCS, suggesting a synergistic flame-retardant effect between PCS and MXene. Moreover, the layered blocking effect of MXene nanosheets and the synergistic coking effect of MXene and PCS produced dense carbon protective layers, achieving remarkable smoke suppression (Figure 9b). Liu et al. [81] grew zirconium aminotrimethylphosphonate (Zr-AMP) in situ on the surface of MXene nanosheets (Zr-MXene) and then dispersed MXene uniformly in a TPU matrix via solution blending. TPU exhibited a low limited oxygen index of 21.3%, while the TPU nanocomposite with 2 wt% Zr-AMP and MXene/TPU with filler of the same content exhibited equal indices of 24.0%. Surprisingly, the limited oxygen index of TPU nanocomposites containing 2 wt% Zr-MXene reached 24.5%, this being higher than that of MXene and Zr-AMP nanocomposites at the same content. This result indicates the synergistic flame-retardant effect between MXene and Zr-AMP.

## 5. Conclusions and Outlook

The development of MXene/elastomer nanocomposites is still in its infancy, but the nanocomposite performances in areas such as EMI shielding and flexible sensors are outstanding. With flexible and highly elastic elastomer matrices, the properties of MXene can be better exploited for a wider range of applications. In this review, we have described and analyzed preparation methods for MXene, including HF etching, in situ HF-forming etching, and mixed-acid etching. The preparation methods for MXene/elastomer nanocomposites have also been presented, including latex blending, solution blending, and matrix backfilling. The excellent performances and diverse applications of MXenes and elastomers (e.g., NR, SBR, NBR, PDMS, TPU) in mechanics, EMI shielding, flexible sensors, energy storage and conversion, and flame retardants have also been discussed. The synergy and functionality between elastomers and MXenes to achieve high conductivity, hydrophilicity, surface functional groups, and layered structures have raised elastomer nanocomposites to a higher level.

MXene/elastomer nanocomposites still face challenges in practical applications. First, MXene faces many problems regarding preparation. These problems include hazards and contamination by HF, the unstable performance and quality of MXene nanosheets, difficult long-term preservation owing to easy oxidation, and high production costs. Second, to produce MXene/elastomer nanocomposites with outstanding performances, excellent dispersion of MXenes should be achieved or nanocomposite structures should be constructed and these processes hinder preparation. Finally, current research on MXene/elastomer nanomaterials is focused on achieving high performances, while studies on basic mechanisms are scarce. Once these problems are addressed, we believe that MXene/elastomer nanocomposites will demonstrate great performances and have favorable application prospects.

## Data Availability

Not applicable.
